# Supplementary material for: Unfolded Protein Response Is Activated by Aurora Kinase A in Esophageal Adenocarcinoma
Source: Cancers (Basel). 2022 Mar 9;14(6):1401. doi: 10.3390/cancers14061401 (PMC8946061; doi:10.3390/cancers14061401)
Supplement: Supplementary file 1 [file cancers-14-01401-s001.zip › cancers-1594628-supplementary.pdf]

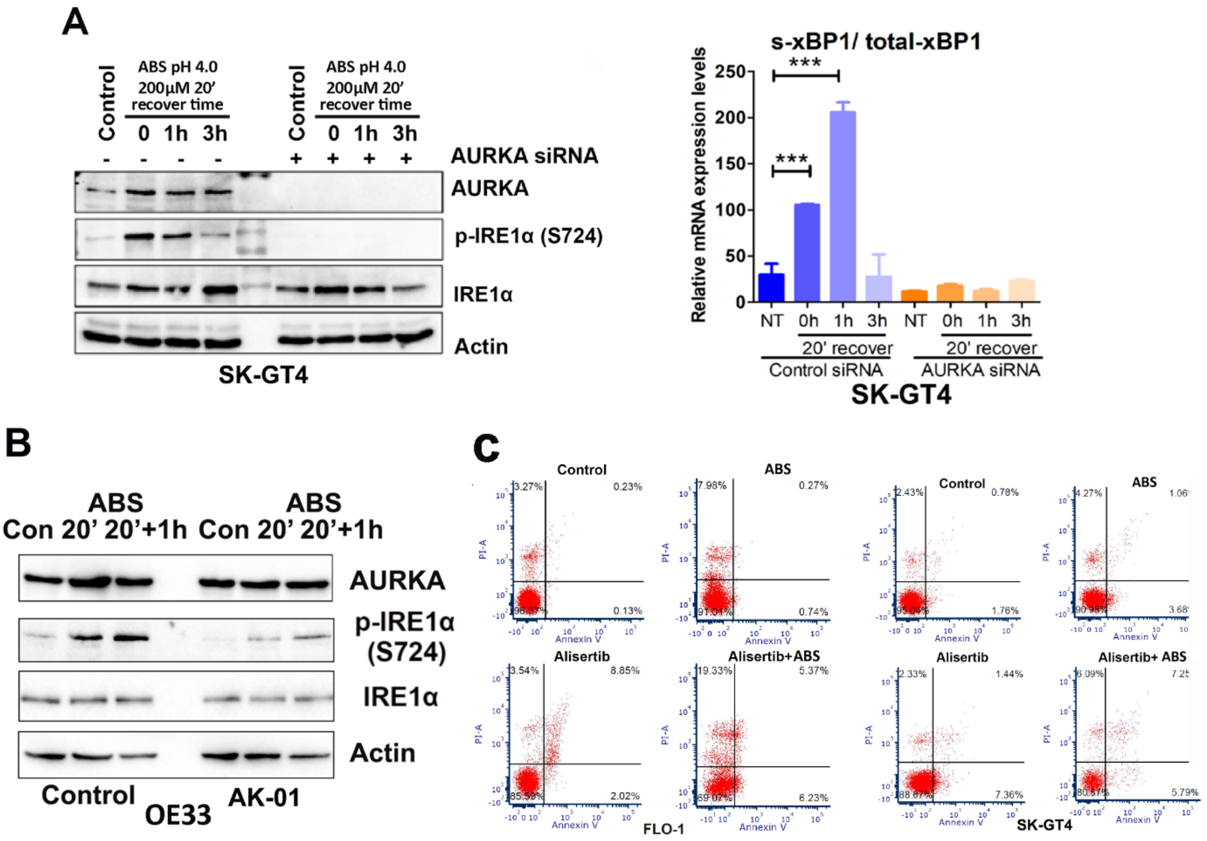

Supplementary Figure 1

Figure S1. AURKA promotes ABS induced IRE1α phosphorylation. A) Left panel: Western blot analysis of AURKA, p-IRE1α (S724), IRE1α, and β-actin protein expression in control siRNA or AURKA siRNA transfected FLO-1 cells with control or ABS (pH4.0, 200μM, 20') exposure followed by recovery at indicated time courses (0, 1h or 3h). Right panel: qRT-PCR analysis of the ratio of spliced-xBP1 (s-xBP1)/ total-xBP1 in the same cells as A left panel. B) Western blot analysis of AURKA, p-IRE1α (S724), IRE1α, and β-actin protein expression in OE33 cells with or without AK-01 (200nM, 48h), followed by ABS (pH4.0, 200μM, 20') exposure with indicated time courses of recovery (0, 1h). C) Left panel: Flow cytometry analysis of Annexin V and PI staining in FLO-1 cells with ABS (pH4.0, 200μM, 20') exposure followed by 3h recovery, at 72 hours after control siRNA or AURKA siRNA transfection. Right panel: similar results in SK-GT4 cells as the left panel *** $p < 0.001$.

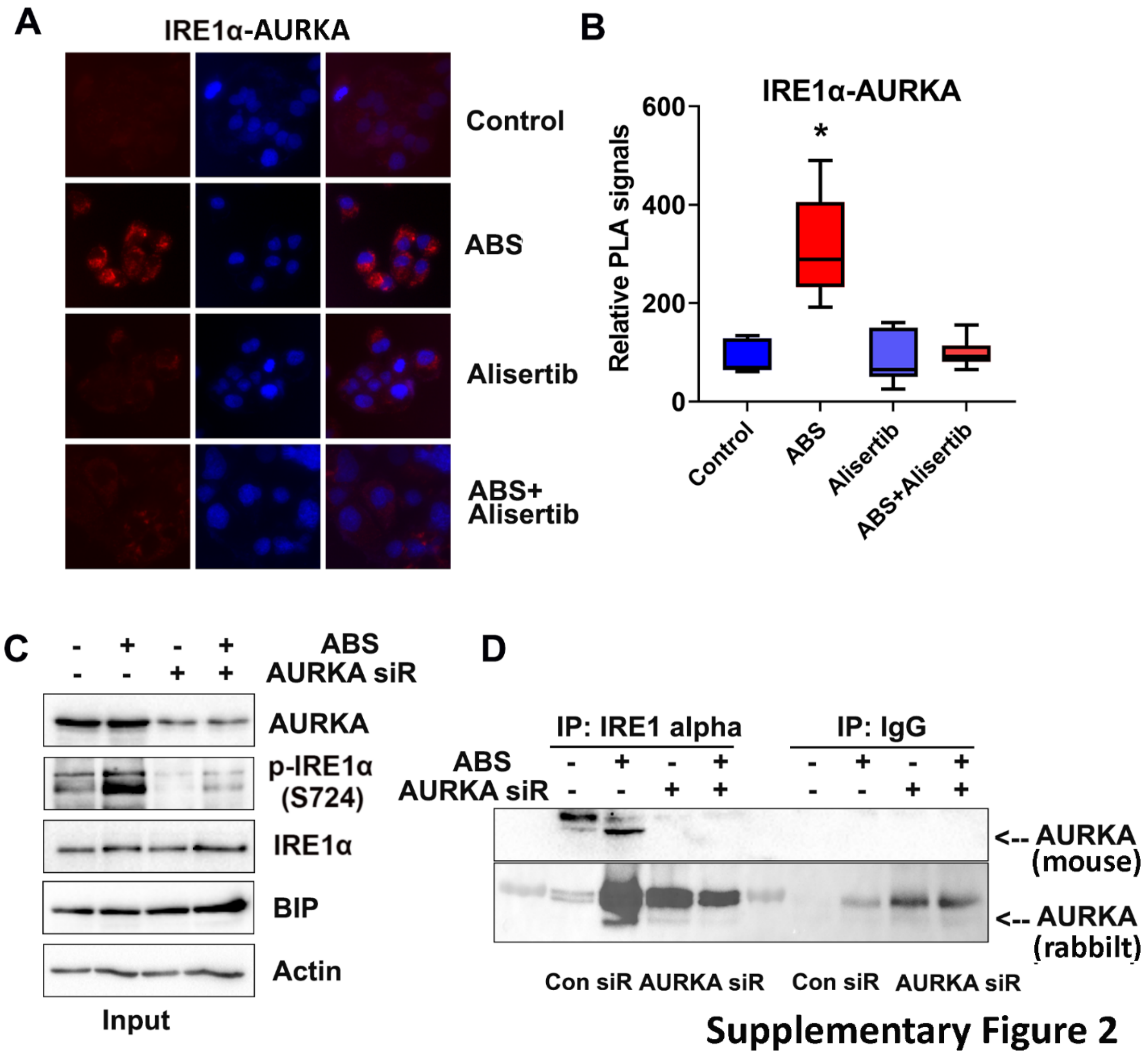

Figure S2. AURKA is co-localized and bond to IRE1α. A) *In situ* proximity ligation assay (PLA) Immunofluorescence staining in FLO-1 control or alisertib 200nM 48h treated cells with or without ABS (pH4.0, 200μM, 20′) exposure at the last 20′ before cell harvest. The red signal indicates the positive PLA signal. B) Quantification of A using ImageJ software. C) Western blot analysis of AURKA, p-IRE1α (S724), IRE1α, BIP, and β-actin protein expression in IRE1α IP input samples of FLO1 cells. Cells were transfected by control or AURKA siRNA for 72h with or without ABS (pH4.0, 200μM, 20′) exposure at the last 20′ before cell harvest. D) Western blot analysis of AURKA in IRE1α IP or IgG IP samples from C. *P<0.05.

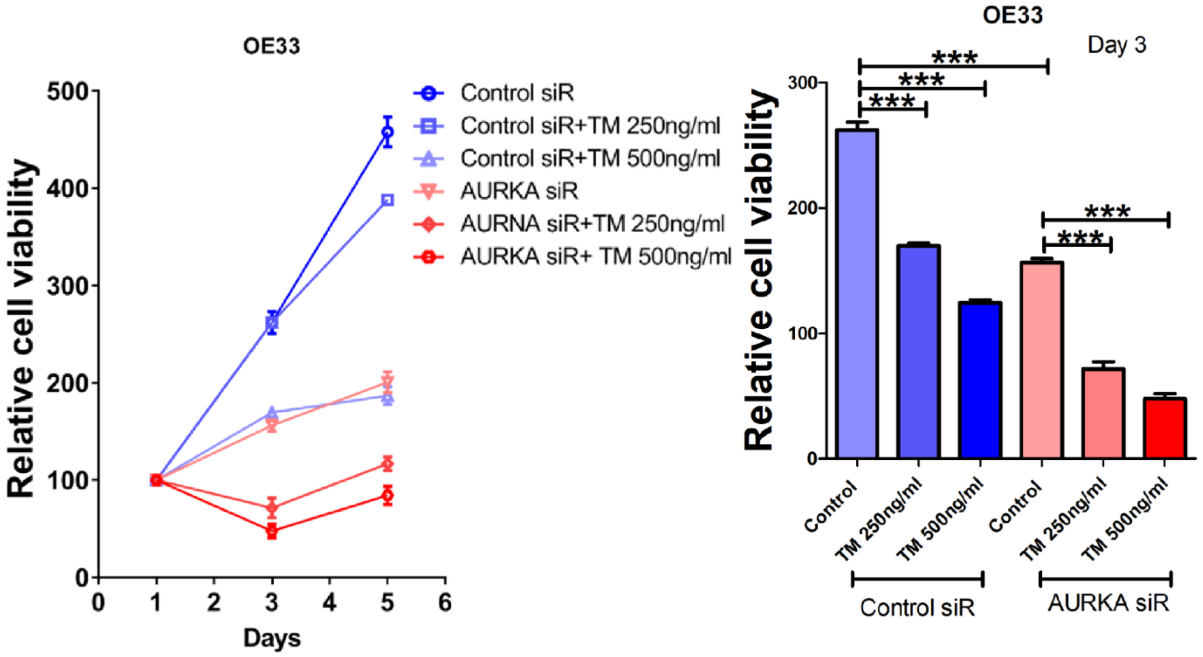

Supplementary Figure 3

Figure S3. AURKA siRNA knockdown promotes EAC cells to Tunicamycin treatment. Left panel: ATP-Glo analysis of control siRNA or AURKA siRNA transfected OE33 cells with Tunicamycin (TM) 250 ng/ml or 500 ng/ml for three days or five days. Right panel: Cell viability analysis of day 3 TM treatment from left panel, *** $p < 0.001$.

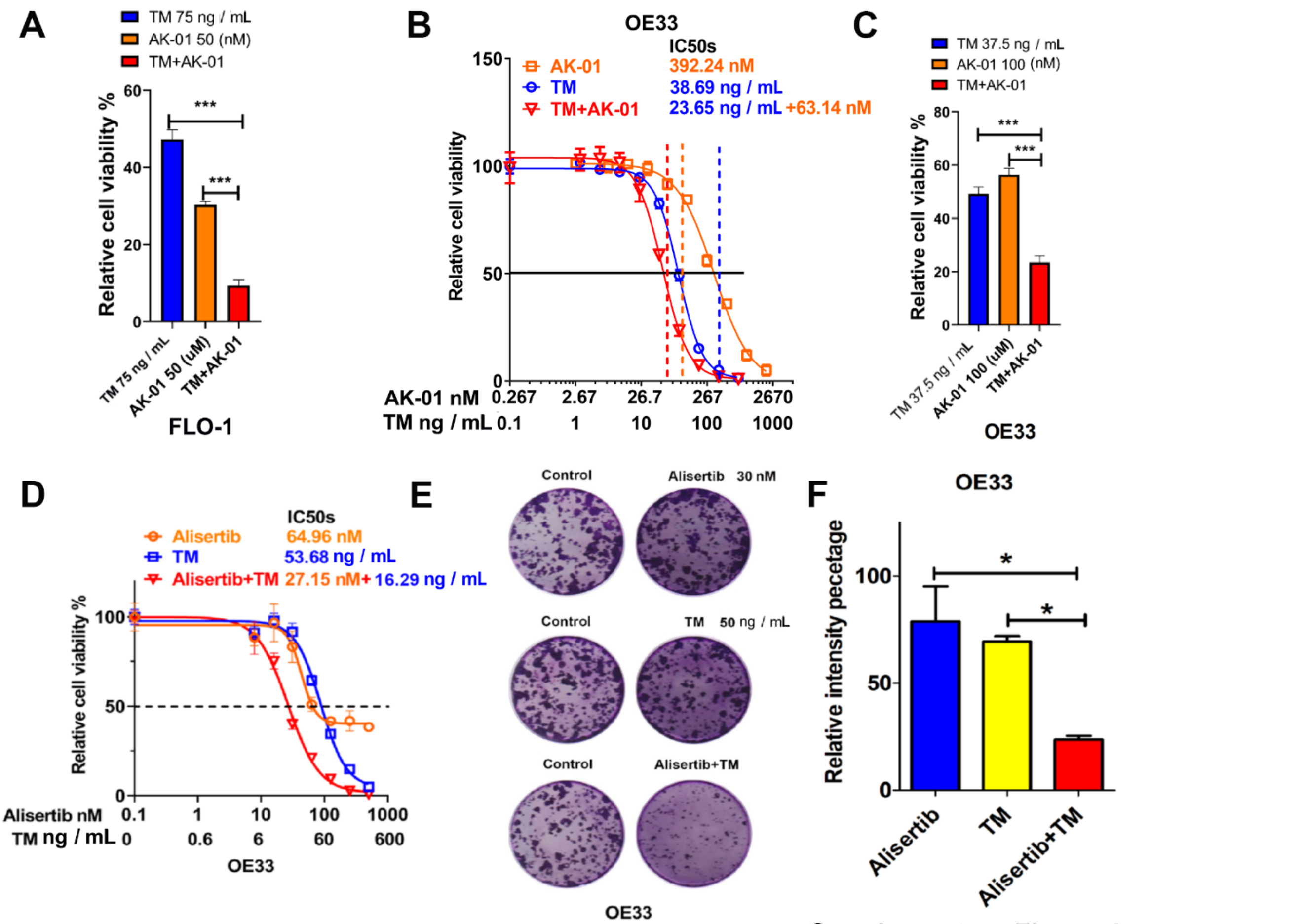

Supplementary Figure 4

Figure S4. AURKA inhibition synergizes with Tunicamycin in EAC cells. A) Cell viability of TM (75 ng/ml) or AK-01 (50 nM) showed the most significantly synergistic effect of the combination treatment in Figure 6B. B) ATP-Glo analysis of FLO-1 cells treated with Tunicamycin, AURKA inhibitor AK-01, or combination for five days. C) Cell viability of one selected drug concentration showed the most significantly synergistic effect of the combination treatment in B. D) Similar results as in Figure 6C in OE33 cells. E) Representative wells of clonogenic cell survival assay in FLO-1 and OE33 cells treated with Tunicamycin, AURKA inhibitor Alisertib, or the combination. F) Quantification of E, *P<0.05, *** $p < 0.001$.

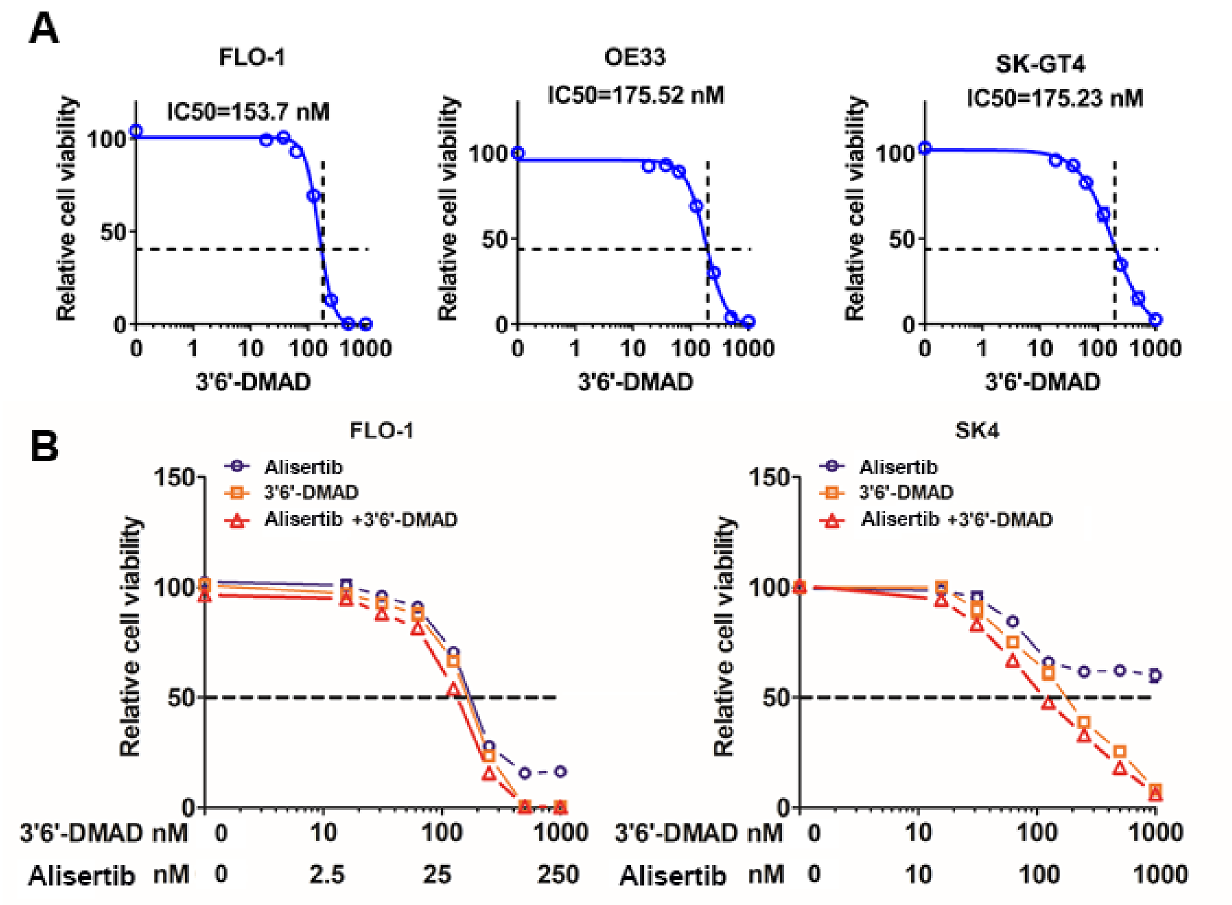

Supplementary Figure 5

Figure S5. AURKA inhibition shows no synergistic effect with IRE1α inhibitor. A) ATP-Glo analysis of FLO-1, OE33, and SK-GT4 cells treated with IRE1α inhibitor 3′6′-DMAD. B) ATP-Glo analysis of FLO-1 and SK-GT4 cells treated with 3′6′-DMAD, Alisertib, or the combination.

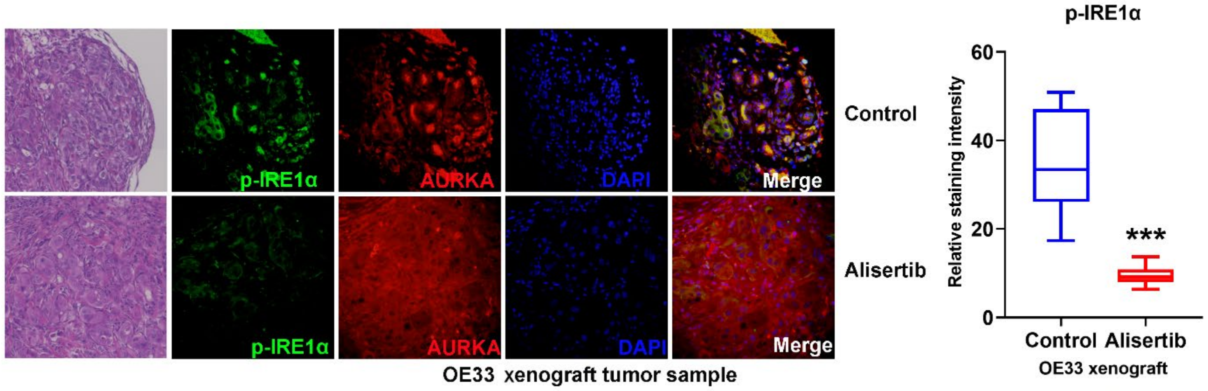

Supplementary Figure 6

Figure S6. P-IRE1α and AURKA protein are co-localized in FLO-1 xenograft tumor samples. Left panel: H&E staining (left panels) and IF staining of p-IRE1α (S724, green signal), AURKA (red signal) in FLO-1 xenograft tumor samples with or without alisertib treatment. 200x magnification. Right panel: Quantification of p-IRE1α staining in the left panel using ImageJ.

Supplementary tables

Table S1. Age, sex, and histology information of human esophageal tissue samples.

Sample ID	Sex	Age	Site	Pathology	Sample ID	Sex	Age	Site	Pathology
1	M	68	GEJ	ADENO	33	M	61	ESO	NORMAL
2	M	71	ESO	ADENO	34	M	65	ESO	NORMAL
3	F	50	GEJ	ADENO	35	F	65	ESO	NORMAL
4	M	66	GEJ	ADENO	36	M	56	ESO	NORMAL
5	M	77	ESO	ADENO	37	missing	missing	ESO	NORMAL
6	M	56	GEJ	ADENO	38	F	66	ESO	NORMAL
7	M	72	GEJ	ADENO	39	M	53	ESO	NORMAL
8	M	73	ESO	ADENO	40	M	60	ESO	NORMAL
9	M	66	GEJ	ADENO	41	M	59	ESO	NORMAL
10	M	62	ESO	ADENO	42	F	57	ESO	NORMAL
11	missing	missing	ESO	ADENO	43	M	52	ESO	NORMAL
12	F	51	ESO	ADENO	44	M	58	ESO	NORMAL
13	M	71	ESO	ADENO	45	M	44	ESO	NORMAL
14	M	61	ESO	ADENO	46	M	73	ESO	NORMAL
15	missing	51	ESO	SCQ	47	F	45	ESO	NORMAL
16	missing	65	GEJ	ADENO	48	missing	53	ESO	NORMAL
17	M	61	ESO	SCQ	49	missing	69	ESO	NORMAL
18	M	65	GEJ	ADENO	50	missing	66	ESO	NORMAL
19	F	65	ESO	ADENO	51	F	61	ESO	NORMAL
20	M	56	ESO	SCQ	52	M	62	ESO	NORMAL
21	F	missing	ESO	ADENO	53	M	56	ESO	NORMAL
22	F	66	ESO	ADENO	54	F	40	ESO	NORMAL
23	M	53	ESO	SCQ	55	F	79	ESO	NORMAL
24	M	60	ESO	ADENO	56	M	53	ESO	NORMAL
25	M	59	ESO	SCQ	57	F	55	ESO	NORMAL
26	F	57	ESO	SCQ	58	M	77	ESO	NORMAL
27	M	52	GEJ	ADENO	59	M	66	ESO	NORMAL
28	M	58	ESO	SCQ	60	M	69	ESO	NORMAL
29	M	44	ESO	SCQ	61	F	46	ESO	NORMAL
30	M	58	GEJ	ADENO	62	M	68	ESO	NORMAL
31	M	79	GEJ	ADENO					
32	F	68	GEJ	ADENO					

ESO: esophagus, GEJ: esophagogastric junction, ADENO: adenocarcinoma, SCQ: Squamous Cell Carcinoma.

Table S2. Primers for q-RT-PC.R

Gene	Forward	Reverse
**AURKA**	GAGGTCCAAAACGTGTTCTCG	ACAGGATGAGGTACACTGGTTG
**BIP**	CATCACGCCGTCCTATGTCG	CGTCAAAGACCGTGTTCTCG
**s-XBP1**	CTGAGTCCGAATCAGGTGCAG	ATCCATGGGGAGATGTTCTGG
**T-XBP1**	TGGCCGGGTCTGCTGAGTCCG	ATCCATGGGGAGATGTTCTGG
**IRE1α**	CACAGTGACGCTTCCTGAAAC	GCCATCATTAGGATCTGGGAGA

Abbreviations

EAC: esophageal adenocarcinoma
NE: normal esophagus
AURKA: Aurora kinase A
UPR: Unfolded protein response
ER: endoplasmic reticulum
TCGA: The Cancer Genome Atlas
GEO: Gene Expression Omnibus
qRT-PCR: Real-Time Quantitative Reverse Transcription
IF: immunofluorescence
IP: immunoprecipitation
IHC: immunohistochemistry
PLA: proximity ligation assays

BiP: binding immunoglobulin protein
BE: Barrett's esophagus
ABS: acidic bile salts
GERD: Gastroesophageal reflux disease
IRE1α: serine/threonine-protein kinase/endoribonuclease inositol-requiring enzyme 1 α
p-IRE1α: phosphor-IRE1α
PERK: PKR-like ER kinase
ATF6: activating transcription factor 6
CHTN: National Cancer Institute Cooperative Human Tissue Network
BSA: bovine serum albumin
GSEA: Gene Set Enrichment Analysis
DCA: deoxycholic acid
NGS: Next Generation Sequencing
TM: Tunicamycin
HGD: high-grade dysplasia
c-PARP: cleaved-PARP
s-xBP1: spliced-xBP1
